# Healthcare simulation—Past, Present, and Future

**DOI:** 10.1097/j.pbj.0000000000000270

**Published:** 2024-10-23

**Authors:** Abel Nicolau, Joana Berger-Estilita, Willem L. van Meurs, Vitor Lopes, Marc Lazarovici, Cristina Granja

**Affiliations:** a Simulation Center (SIMFMUP), Faculty of Medicine, University of Porto, Porto, Portugal; b CINTESIS@RISE, Faculty of Medicine, University of Porto, Porto, Portugal; c Institute of Anaesthesiology and Intensive Care, Salem Spital, Hirslanden Hospital Group, Bern, Switzerland; d Institute for Medical Education, University of Bern, Bern, Switzerland; e Department of Obstetrics-Gynecology and Pediatrics, Faculty of Medicine, University of Porto, Porto, Portugal; f Department of Surgery and Physiology, Faculty of Medicine, University of Porto, Porto, Portugal; g Department of General Surgery, São João University Hospital Center, Porto, Portugal; h Institute for Emergency Medicine and Management in Medicine—INM, LMU University Hospital, Munich, Germany

In acute care settings, quick decision making and rapid execution are critical. Chesley Sullenberger's emergency landing of Flight 1549 in 2009, where he saved 155 lives,^1^ epitomizes this. Similar scenarios in medicine include the urgent need to choose between open and endovascular repair in an abdominal aortic aneurysm or between cardiopulmonary resuscitation (CPR) and cesarean delivery during obstetric cardiac arrest.

Such high-stakes decision making underpins the evolution of health care simulation, transitioning from passive dummies and large mammals to advanced patient simulators and simulated patients (actors). This shift allows for controlled andragogical content delivery and accelerated experiential learning. The concept of fidelity—functional and physical—plays a crucial role in the effectiveness of these simulations. Functional fidelity resembles trainee behavior in simulated and real environments. By contrast, physical fidelity involves the match between the appearance and behavior of the simulated and real patients.

Physiological fidelity is essential in acute care simulations requiring real-time, realistic intervention responses. Mathematical human physiology and pharmacology models facilitate these simulations,^2^ similar to the necessity of flight models in aviation simulators. Innovations in simulation include interactive visualizations and objective performance metrics based on physiological parameters.

The transition from a fixed duration/variable performance model to a fixed performance/variable duration paradigm in medical training is underway. With the ability to simulate multiple critical scenarios within a short period, the learning experience has become more efficient and comprehensive. Despite these advancements, challenges remain in ensuring that simulations are as realistic and effective as possible. Continuous improvement in simulation technology and methods is essential for maintaining high health care education and training standards.

The teaching of surgery and other skills has historically followed the “see one, do one, teach one” method established by Halsted.^3^ However, this traditional approach has challenged various factors, including restricted working hours, economic and social contexts, productivity, legal and ethical issues, and time constraints.^4^

The advent of laparoscopy and the necessity for quick education in minimally invasive techniques brought simulation to the forefront.^5^ While effective for laparoscopic surgery, simulation's role in open surgery remains underexplored.^6^ Similarly, the heterogeneity in robotic surgery curricula highlights the need for standardized training methods.^7^

The COVID-19 pandemic further disrupted traditional surgical training, limiting residents' exposure to operating rooms. This shift necessitated the adoption of e-learning and new technologies such as virtual and augmented reality.^8^ These tools have potential but require further integration and validation within surgical curricula.^9^

Our augmented reality projects demonstrate the potential of telementoring in surgical education. This technology facilitates training students and residents through a 3-phase educational model that challenges the traditional mentor–mentee relationship. Initial results indicate effectiveness, suggesting a need for deliberate practice and motivation within surgical training curricula.^10^

Integrating augmented reality (AR) and virtual reality (VR) into surgical training is a significant step forward. These technologies provide immersive, interactive experiences that enhance learning outcomes. For example, AR can overlay digital information onto real-world environments, aiding in complex surgical procedures by providing real-time data and guidance. Conversely, VR can simulate entire surgical environments, allowing trainees to practice procedures risk-free.

Despite their promise, these technologies face several challenges in implementation. High costs, technical complexities, and the need for specialized training for both instructors and learners are significant barriers. In addition, comprehensive studies are needed to validate the effectiveness of AR and VR in improving surgical skills and patient outcomes.

Collaborative efforts among academic institutions, health care organizations, and technology developers are crucial to address these challenges. Developing standardized protocols and guidelines for AR and VR in surgical training can help streamline their integration into curricula. Moreover, ongoing research and innovation are essential to refine these technologies and maximize their educational potential.

Several pressures, including remote areas, mass casualty events, and the COVID-19 pandemic, have highlighted the need for innovative solutions in health care delivery. While technology offers alternatives, its possibilities and limitations must be understood. The concept of eXtended Reality (XR) encompasses AR, VR, and mixed reality (MR), providing various enhancements to real-world environments.

XR technologies can significantly improve education and health care services. Applications range from adding sensory feedback to simulated patients and training procedures in safe environments, to fully immersive virtual worlds for remote teamwork and training. These technologies offer new perspectives but pose challenges in proper implementation and utilization.^11^

Telemedicine can also benefit from XR, with real-time 3D reconstructions of clinical environments allowing remote experts to interact naturally with on-site practitioners. This experimental approach has shown promise and could shape future developments.

However, these tools are only as effective as the professionals using them are. The transition from virtual skills to real-world practice requires thorough research and validation. Continued advancement in the field, paired with robust educational research, is essential.

One key advantage of XR technologies is their ability to create highly realistic simulations that closely mimic real-world clinical scenarios. This realism enhances the learning experience, making it easier for trainees to transfer skills acquired in simulations to actual clinical practice. For instance, XR can simulate a clinical environment's sounds, sights, and smells, providing a comprehensive sensory experience.

Moreover, XR technologies enable remote learning and collaboration, breaking geographical barriers and making high-quality training accessible to health care professionals worldwide. This is particularly beneficial in remote or underserved areas with limited access to advanced training facilities. By leveraging XR, health care organizations can provide practitioners with continuous education and support, regardless of location.^12^

Despite these benefits, the successful integration of XR into health care education requires careful planning and consideration. Robust frameworks and guidelines must be developed to ensure the effective and ethical use of these technologies. In addition, investing in the training of educators and technicians is crucial to maximizing the potential of XR in health care simulation.

Health care simulation is crucial for clinical education, providing a safe skill and decision-making practice environment. It spans basic procedures to complex emergencies, promoting competence, confidence, and error management. Standardization ensures consistency and quality in training, necessitating scenario design, assessment, and debriefing guidelines. Training for simulation professionals is essential for maintaining standards.^13^

The European Master in Healthcare Simulation (EuroHealthSim) aims to foster collaboration among diverse European simulation centers. The curriculum includes core aspects of clinical education; simulation technology; research methodologies; and cutting-edge subjects such as AI, robotic surgery, and telesimulation. This program aspires to be a transformative force in simulation education, anticipating future advancements in health care and education.

EuroHealthSim coordinated by the FMUP in partnership with LMU Munich and MUNI MED in Brno, Czech Republic, seeks to establish a sustainable European Master in Healthcare Simulation. Its comprehensive approach and global recognition aim to enhance simulation education and health care delivery quality in Europe and beyond.

The EuroHealthSim program addresses several key areas to ensure its success and impact on health care education. First, it emphasizes the importance of interdisciplinary collaboration, bringing experts from various fields together to develop a well-rounded curriculum. This collaboration fosters innovation and ensures that the program remains relevant in an ever-evolving health care landscape.

Second, EuroHealthSim strongly emphasizes research and development. By encouraging students and faculty to engage in cutting-edge research, the program aims to advance health care simulation technologies and methodologies. This research component is vital for continuously improving the quality and effectiveness of simulation-based education.

Third, the program incorporates a global perspective, recognizing the diverse health care needs and challenges across different regions. By collaborating with international partners and institutions, EuroHealthSim aims to create a culturally sensitive and adaptable curriculum that can be applied in various health care settings worldwide.

Furthermore, EuroHealthSim prioritizes the development of soft skills, such as communication, teamwork, and leadership, which are essential for health care professionals. Through simulation-based training, students can practice and refine these skills in a controlled environment, preparing them for real-world interactions with patients and colleagues.

To ensure the program's sustainability and scalability, EuroHealthSim is committed to continuous evaluation and improvement. Regular assessments and feedback from students, faculty, and industry partners will help identify areas for enhancement and ensure that the program remains at the forefront of health care simulation education.

The crucial role of simulation in health care education and training is highlighted. From its historical evolution to current practices and future directions, simulation provides a vital platform for developing and refining health care skills in a controlled, safe environment. Integrating new technologies, such as XR and telemedicine, promises to enhance educational outcomes and health care delivery further.

As we look to the future, the continued advancement of simulation-based education and the establishment of programs such as EuroHealthSim will play a pivotal role in meeting the evolving needs of health care professionals. The commitment to research, innovation, and collaboration will ensure that simulation remains a cornerstone of health care education, ultimately improving patient care and safety.

Simulation in health care has come a long way from its humble beginnings. The evolution from basic manikins to sophisticated, high-fidelity simulators has transformed how health care professionals are trained. These advancements have not only improved the quality of education but also have significantly influenced patient safety and outcomes.

Looking ahead, the future of health care simulation is bright. Integrating emerging technologies, such as artificial intelligence, machine learning, and big data analytics, holds great promise. These technologies can enhance the realism and effectiveness of simulations, providing personalized learning experiences tailored to the needs of individual learners.

Moreover, adopting a more holistic approach to health care education, which includes technical skills and emotional and psychological aspects, will further enhance the impact of simulation. Addressing issues such as burnout, empathy, and communication through simulation can help create well-rounded health care professionals better equipped to handle the complexities of modern health care.

Another exciting development is the increasing collaboration between academic institutions, health care organizations, and industry partners. These collaborations foster innovation and enable the development of cutting-edge simulation technologies and methodologies. By working together, stakeholders can pool resources, share expertise, and drive the field forward.

Furthermore, expanding simulation-based education to include interprofessional training is a significant step forward. Simulation can promote teamwork, collaboration, and a better understanding of each other's roles by training health care professionals from different disciplines together. This interdisciplinary approach is crucial for improving patient care and outcomes in real-world settings.

In conclusion, health care simulation has proven to be an invaluable tool in medical education and training. Over the past 2 decades, its evolution has been remarkable, and the future holds even greater potential. By embracing new technologies, fostering collaboration, and addressing the holistic needs of health care professionals, simulation can continue to play a vital role in shaping the future of health care education and improving patient care worldwide.
